# PD‐L1 expression, CD8+ and CD4+ lymphocyte rate are predictive of pathological complete response after neoadjuvant chemoradiotherapy for squamous cell cancer of the thoracic esophagus

**DOI:** 10.1002/cam4.2359

**Published:** 2019-08-20

**Authors:** Matteo Fassan, Francesco Cavallin, Vincenza Guzzardo, Andromachi Kotsafti, Melania Scarpa, Matteo Cagol, Vanna Chiarion‐Sileni, Luca Maria Saadeh, Rita Alfieri, Ignazio Castagliuolo, Massimo Rugge, Carlo Castoro, Marco Scarpa

**Affiliations:** ^1^ Department of Medicine, Pathology Unit University of Padova Padua Italy; ^2^ Independent statistician Solagna Italy; ^3^ Department of Advanced Traslational Research Veneto Institute of Oncology (IOV‐IRCCS) Padua Italy; ^4^ Department of Oncological Surgery Veneto Institute of Oncology (IOV‐IRCCS) Padua Italy; ^5^ Department of Oncology Veneto Institute of Oncology (IOV‐IRCCS) Padua Italy; ^6^ Department of Molecular Medicine University of Padova Padua Italy; ^7^ Department of Upper GI Surgery Humanitas Research Hospital‐Humanitas University Rozzano Italy; ^8^ General Surgery Unit Azienda Ospedaliera di Padova Padova Italy

**Keywords:** esophageal cancer, induction chemoradiotherapy, neoadjuvant therapy, survival analysis, squamous cell carcinoma

## Abstract

**Background:**

Neoadjuvant chemoradiotherapy (CTRT) can effectively downstage esophageal squamous cell carcinoma (SCC) in patients with locally advanced disease and prolonged survival have been observed in patients with a pathological complete response (ypCR).

**Aims and methods:**

This exploratory study aimed to identify immunological predictors of pCR after neoadjuvant CTRT within SCC microenvironment. The tumor regression after neoadjuvant therapy was measured according to the Mandard score system. Eighty‐eight consecutive patients with SCC of the thoracic esophagus who received neoadjuvant CTRT were included in this retrospective study. Inclusion criteria were neoadjuvant CTRT and the availability of representative histological samples taken at diagnosis. We investigated immunohistochemical expression of CD4, Tbet, FoxP3, CD8, CD80, PD‐L1, and PD‐1, in the pretreatment biopsies and correlated the immunohistochemical profiles to patients’ outcomes.

**Results:**

After neoadjuvant CTRT, 23 patients had pCR, while 65 ones had partial response, stable disease or progression. PD‐L1 expression and CD8+ and CD4+ lymphocyte rate were significantly higher in patients who had ypCR compared to those who had not (10 (0‐55) vs 0 (0‐0), *P *= 0.004, 73 (36‐147) vs 21 (7‐47), *P *= 0.0006 and 39 (23‐74) vs 5 (0‐13), *P* < 0.0001 respectively). The accuracy of expression of PD‐L1+, CD8+, and CD4+ lymphocyte rate in identifying responders was AUC = 0.76 (*P* = 0.001), AUC = 0.81 (*P* = 0.0001) and AUC = 0.75 (*P* = 0.0001), respectively. Within the ypCR group, all patients with high infiltration of CD4+ T cell recurred/relapsed while only the 38.9% of those with low CD4+ T cell infiltration did the same (*P* = 0.058).

**Conclusions:**

PD‐L1 expression and CD8+ and CD4+ lymphocyte rate were predictive of ypCR after neoadjuvant CTRT for SCC of the thoracic esophagus with adequate accuracy. Furthermore, recurrence/relapse was associated with high level of CD4+ T cell infiltration. However, the small sample size prevented to draw definitive conclusions; further studies are necessary to evaluate the prognostic role of these markers.

## Graphical abstract

Mucosal expression of PDL1 and CD8+ and CD4+ lymphocyte rate is predictive of clinical complete response after neoadjuvant CT‐RT for SCC of the thoracic esophagus with adequate accuracy. Furthermore, recurrence/relapse after complete response is associated with high level of CD4+ T cell infiltration.

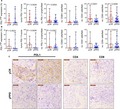

## 1 BACKGROUND

Esophageal cancer is the eighth most common cancer and is the sixth cause of cancer‐related death in US.^1^ Even in locally advanced disease treated with curative intent, 5‐year survival rate remains unsatisfactory varying from 15% to 39%^2^ and esophagectomy accounts of up to 35% of curative resections, despite a high incidence of complications.^3^, ^4^, ^5^ Neoadjuvant therapy (chemotherapy or radio‐chemotherapy) followed by surgical resection improved the outcome, both in short‐ and long‐term.^6^, ^7^ Moreover, neoadjuvant chemoradiotherapy (CTRT) can lead to tumor downstaging and increase the R0 resection rate.^8^, ^9^ This is even more evident in patients presenting locally advance disease.^10^, ^11^ When a pathological complete response (ypCR) is obtained, the increase in survival rate becomes much more evident.^12^, ^13^, ^14^ In the CROSS trial, investigating the result of surgical resection alone compared to surgery after neoadjuvant CTRT, the ypCR rate reached 29% of the included patients.^15^ Currently, based on these data, patients with squamous cell carcinomas (SCCs) of the cervical esophagus who achieve clinical complete response are operated on only in case of recurrence.^16^, ^17^ Nonetheless, it is still not clear how to manage ycCR SCCs of the mid thoracic esophagus. Many efforts has been made to investigate the different outcome of CTRT alone compared to CTRT followed by surgery but only in part focused on CR^18^, ^19^ and many questions remain still unanswered.^20^, ^21^, ^22^, ^23^ In a previous retrospective study, we concluded that new and more accurate protocols are needed to plan a treatment roadmap for ypCR after neoadjuvant CTRT.^24^

In the colorectal cancer, the immunologic landscape of the mucosa is associated with the pathological evidence of early metastatic invasion and with patients' survival,^25^ being a better prognostic predictor than the histopathological features of the tumor.^26^ In esophageal cancer, the expression of the costimulatory molecules CD80 is significantly downregulated and is inversely correlated with TGF‐*β*1 and IL‐10 expression.^27^, ^28^ Moreover, metastatic esophageal cancer cells are less sensitive to specific cytotoxic lymphocytes^29^ and in the upper gastrointestinal cancers CD3+ and CD8+ tumor infiltrating lymphocytes (TILs) show functional exhaustion and express high levels of PD‐1.^30^ These are the main reasons that lead to immune escape of esophageal cancer cells and might be one of the main potential markers of neoadjuvant therapy failure.^31^ Furthermore, in a recent article, several immune‐related genes significantly associated with patients' overall survival and in particular three independent factors (i.e. *ABL1*, *CD38* and *ICOSLG*) have been identified. Validation by immunohistochemistry staining suggested that combination with tumor‐infiltrated CD4+ and CD8+ T lymphocytes would yield higher performance of these predictors in distinguishing cases as high‐ or low‐risk of unfavorable prognosis.^32^ Finally, Thar Min et al. demonstrated that PD‐L1 expression was upregulated in mesenchymal type tumors of esophageal SCC, thus allowing T cell apoptosis in patients with advanced cancer.^33^

This exploratory study aimed to identify, by immunohistochemical profiling, immunological predictors of ypCR after neoadjuvant CTRT within the tumor microenvironment of the esophageal SCC samples obtained at diagnostic endoscopy. In addition, we sought possible predictors of cancer persistence/recurrence in presence of a clinical yCR.

## 2 METHODS

### 2.1 Study design

This is a retrospective exploratory study to inform a validation study. The study was performed according to the REMARK guidelines.^34^ We investigated the immunohistochemical expression of CD4, Tbet, FoxP3, CD8, CD80, PD‐L1, and PD‐1 in the pretreatment endoscopic biopsies as possible predictors of complete response after CTRT for locally advanced esophageal SCC. We evaluated all the consecutive patients presenting in our tertiary referral Centre with SCC of the thoracic esophagus from 1 January 1992 and 31 December 2007 for inclusion. Inclusion criteria were neoadjuvant CTRT and availability of histological samples taken at diagnosis in the archives of the Surgical Pathology Unit of Padua University. Patients presenting systemic disease were excluded, except for those with metastatic celiac lymph nodes. Patients with SCC of the cervical esophagus were excluded as well as those who received neoadjuvant chemotherapy or radiotherapy only. Once neoadjuvant CTRT was completed, surgical resection was offered to patients. The clinical end points were pathological complete response and recurrence after ypCR. PD‐1 and PD‐L1 (immune check point), CD8 (cytotoxic lymphocyte), CD4 (T helper), CD80 (antigen presenting cell costimulatory molecule), Fox‐P3 (T‐reg marker), and Tbet (Th1 subpopulation marker) were the markers chosen to analyze the tumor microenvironment. Immunohistochemistry analysis of tumor immune infiltration on the SCC samples obtained at diagnosis (pretreatment biopsies) was compared according to the response to neoadjuvant CTRT, ypCR vs pathological partial down‐staging (yPPD). All the patients gave written informed consent to the data collection and analysis; the study was performed in accordance with the principles of the Declaration of Helsinki. The Ethics Committee of the Veneto Institute of Oncology (IOV‐IRCCS, Padua, Italy) approved the study (internal code PIRCCE; number 2017/32).

### 2.2 Clinical pretreatment evaluation

When we revised the patients' records, we restaged all the TMN classification according to the seventh edition in order to have comparable data among the different periods. (Union for International Cancer Control).^35^ Before the treatment, every patient underwent esophageal endoscopy with biopsies retrieval and endoscopic ultrasonography, bronchoscopy, upper gastrointestinal tract radiography, CT scan with contrast of neck, chest, and abdomen. From 2005, positron emission tomography scan was included as part of the pretreatment evaluation.

### 2.3 Clinical response assessment

The definition of clinical complete response (ycCR) was: disappearance of the tumor lesion, ulceration, and absence of cancer cells in biopsy specimens upon endoscopic observation of the entire esophagus.^36^, ^37^ The Response Evaluation Criteria in Solid Tumours (RECIST) guideline was used to evaluate the lymph nodes' CR. CT scan and, when in use, PET scan were performed to assess distant metastasis.^38^

### 2.4 Neoadjuvant therapy

Across the time frame considered for the study, different neoadjuvant chemotherapy regimens were adopted. The most common treatment administered consisted of platinum‐derived drugs (commonly cisplatin 100 mg/m^2^) on day 1 in association with 5‐fluorouracil from Day 1 to 5 (1000 mg/m^2^); this scheme was recycled two to three times accordingly to patients' response. Some of the patients received taxanes in association.

Radiotherapy was administered at the same time but, similarly to chemotherapy, the protocols differ along the years. Commonly, a total dose of 45‐50 Gy was administered (1.8 Gy daily) with a target field involving ±5 cm over the tumor extension, ±2 cm over pathologic lymph nodes, mediastinum and supraclavicular fossa. A total dose of 30.6 Gy was administered using anteroposterior‐posteroanterior fields in the initial phase. Subsequently, the radiation portal was extended in order to enclose primary tumor together with pathological lymph nodes including an additional margin of 2 cm employing an oV‐cord conformal oblique weld and reaching a final total dose of 45‐50.4 Gy. Patients not suitable for surgery underwent evaluation for further radiotherapy.

### 2.5 Surgical resection

Surgery was planned from 4 to 6 weeks after the neoadjuvant treatment was completed.^29^ Patients who did not undergo surgery but showed cancer recurrence during follow‐up were evaluated for salvage esophagectomy. Details on the surgical techniques have been published elsewhere.^30^, ^31^ The outpatient clinic follow‐up was set at 1‐3‐6‐12 months postoperatively; after the first year the examination was scheduled every 6‐12 months.

### 2.6 Histology

Pretreatment endoscopy biopsy specimens from each patient were submitted to the Surgical Pathology Unit in an adequate volume of 10% formalin for 8 h and routinely processed according to a standardized local protocol.

Sections (3‐4 μm) from formalin‐fixed and paraffin‐embedded human specimens were stained with hematoxylin and eosin and reevaluated according to WHO 2010 classification of the gastrointestinal tumors by a gastrointestinal pathologist (M.F.), who was unaware of clinical data.

The end point of this study was the presence of ypCR. The tumor regression after neoadjuvant therapy was measured according to the Mandard score system.^39^ The definition used for pathologic complete response (ypCR) to treatment was the presence of fibrosis / fibro‐inflammation with no microscopic evidence of cancer remnant within a gross lesion entirely submitted for evaluation and with no evidence of metastatic cancer cells in lymph nodes.

### 2.7 Immunohistochemistry

Immunohistochemical staining was performed on formalin‐fixed, paraffin‐embedded tissue sections using a fully automated system (Bond™‐maX; Leica, Newcastle Upon Tyne, UK). Briefly, tissue sections were deparaffinized in Bond Dewax Solution (catalog # AR9222; Leica) at 72°C, rinsed in ethanol, and rehydrated in distilled water. Sections were pretreated using heat‐mediated antigen retrieval with sodium citrate buffer (catalog # RE7113‐CE; pH6, Epitope Retrieval Solution 1, Leica) for 30 minutes a 99°C. Specimens were then incubated respectively with PD‐1 (catalog # ab52587; clone NAT105; Abcam, Cambridge, UK; 1:150), PD‐L1 (catalog # M3653; clone 22C3; Dako, Glostrup, Denmark; 1:50), CD4 (catalog # M7310; clone 4B12; Dako; 1:40), CD8 (catalog # PA0183; clone 4B11; Leica; 1:100), B7‐1/CD80 (catalog # MAB140‐100; clone 37711; R&D Systems Minneapolis, MI; 1:100), FoxP3 (catalog # ab20034; clone 236A/E7; Abcam;1:400), T‐bet/Tbx21 (catalog # ab91109; clone 4B10; Abcam;1:200) and detected using the Bond Polymer Refine Detection Kit (catalog # DS9800; Leica) according to the manufacturer's protocols. The staining was visualized with 3,3′‐diaminobenzidine (DAB; catalog #DS9800; Leica) and the slides were lightly counterstained with hematoxylin (catalog #DS9800; Leica). Sections were then dehydrated, cleared, and mounted. Appropriate positive and negative control samples were run concurrently (data not shown).

Slides were evaluated and jointly scored by two pathologists on a Leica DM4000B microscope (Leica Biosystems) and images were acquired by using the Leica Application Suite (LAS version 3.8; Leica) software.

PD‐L1 expression was assessed both in neoplastic epithelia and in infiltrating leukocytes. Three different parameters were considered: (a) PD‐L1 staining intensity in neoplastic cells categorizing cases in high (in presence of staining detected up to 5% of the neoplastic cells) and low (<5%) PD‐L1 expression^40^, ^41^, ^42^; (b) a semiquantitative pathology H‐score, defined as the aggregate of total percentage of tumor cells expressing PD‐L1 at each particular intensity level from 0, +1 (weak intensity), +2 (moderate intensity) or +3 (strong intensity); in brief, the H score was defined as (Percent of PD‐L1 1+ tumor cells multiplied by intensity of 1) + (Percent of PD‐L1 2+ tumor cells multiplied by intensity of 2) + (Percent of PD‐L1 3+ tumor cells multiplied by intensity of 3), and this composite score can range from 0 (a tumor which is completely negative) to a maximum of 300 (a tumor in which all the cells feature a 3+ staining); (c) stromal positive leukocytes were counted in 5 HPF (40×).^43^, ^44^

For the other immunohistochemical markers, positive cells were counted in 5 HPF (40×).

### 2.8 Clinical follow‐up

Follow‐up visits were scheduled every 3 months in the first year after surgery, every 6 months during the next 2 years and every 12 months thereafter. An upper gastrointestinal endoscopy was performed regularly 1 year after surgery or earlier based on the clinical findings, with direct evaluation of the remaining esophagus, anastomosis, and of the esophageal replacement conduct. Functional results were assessed based on clinical and endoscopic findings.

### 2.9 Statistical analysis

Due to the retrospective design of the study and the relatively rarity of the primary end point occurrence, (ypCR) the sample size was not defined a priori and all the consecutive patients who met the inclusion criteria were included. Moreover, a posteriori power calculation was almost pleonastic since if a difference resulted statistically significant the sample size of the two groups was large enough to detect it. Only available data were analyzed, and no imputation was done for missing data. No multivariable models were created due to the small sample size.

Median and interquartile range were used to describe continuous variables. The Mann‐Whitney test to compare continuous variables and the Fisher test was used to study categorical variables. The immunological markers of ypCR were tested in the subgroup of patients with the ROC curve analysis. Since there is no codified threshold value for T cell subpopulation infiltration in esophageal SCC, we dichotomized patients into low‐ or high‐positive cells (CD4+, CD8+, CD80+, Tbet+, FoxP3+) infiltration subgroups according to threshold values obtained from ROC curves analysis. The Kaplan‐Meier estimate was used to perform the survival analysis from the date of the initial diagnosis and the log‐rank test was used to compare the subgroup survival. Due to the small sample size, nonparametric combination test was used to compare immunosurveillance data in case of recurrence. All tests were two‐sided with a p‐value considered statistically significant when less than 0.05. The software used to perform statistical analysis was SAS 9.1 (SAS Institute, Cary, NC) and R 3.5 (R Foundation for Statistical Computing, Vienna, Austria).

## 3 RESULTS

### 3.1 Patient selection

**Figure 1:**
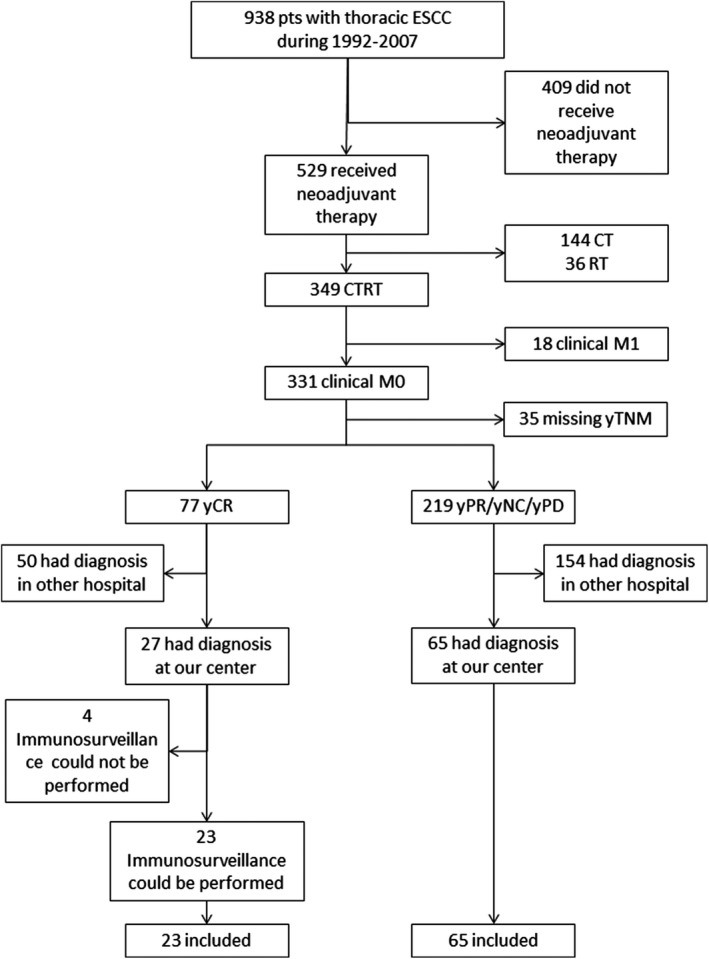
Flowchart of included patients. Abbreviations: PPD: persistence or progressive disease (as opposed to yCR). SCC: esophageal squamous cell carcinoma.

All 938 patients referring for thoracic SCC to the Centre for Oesophageal Diseases located in Padua between 1 January 1992 and 31 December 2007 were retrospectively evaluated using a prospectively collected database. Among 349 patients who received CTRT, 18 were excluded due to clinical M1 stage and 35 due to unavailable restaging after neoadjuvant therapy (yTNM). Two hundred and four patients were referred for treatment of thoracic SCC to our center from other centers, thus the paraffin‐embedded blocks from endoscopic biopsy at diagnosis were not available in our Surgical Pathology archives. Ninety‐two patients had the endoscopy performed in our center at the diagnosis of SCC, thus the biopsy specimens were available for analysis. At histopathologic/immunological reexamination, four paraffin‐embedded blocks presented an inadequate amount of residual material to be further considered in the analysis. Patient selection is shown in Figure 1.

### 3.2 Patient characteristics

Eighty‐eight patients who underwent neoadjuvant therapy for thoracic SCC from 1992 to 2007 were finally included in the analysis. (Figure 1). Twenty‐three (26.1%) patients had ypCR after neoadjuvant therapy while 65 (73.9%) had yPPD (23 (26.1%) yPR, 24 (27.3%) yNC, 18 (20.5%) yPD). Patient characteristics are shown in Table 1. Demographics and comorbidity status were similar in yCR and yPPD groups (Table 1). G1/G2 grading was more frequent in ypCR patients (95.7% in yCR vs 75.4% in yPPD, *P* = 0.03; Table 1). The most frequent CT scheme was DDP + 5FU [68 (77.3%) patients], followed by platinum‐based scheme [11 (12.5%) patients], cetuximab‐based scheme [5 (5.7%) patients] and other schemes [4 (4.5%) patients]. Low and high PD‐L1 expression either on tumor cells or in lymphocytes was observed in 62 (70.4%) vs 25 (28.4%) patients and in 46 (52.3%) vs 41 (46.6%) patients, respectively. Low and high CD80+ cells infiltration was observed in 68 (77.3%) vs 19 (21.5%) patients. Low and high CD4+ and CD8+T cells infiltration was observed in 60 (68.2%) vs 26 (29.5%) patients and in 41 (46.6%) vs 46 (52.3%) patients, respectively.

**Table 1:** Patient characteristics

	yCR	yPPD	*P*‐value
N pts	23	65	—
Response to neoadjuvant therapy	23 yCR	23 yPR 24 yNC 18 yPD	—
Age, years^a^	63 (57‐72)	64 (55‐70)	0.5123
Male:female	19:4	51:14	0.7715
Tumor site:			0.3525
Upper thoracic	12 (52.2)	25 (38.5)	
Middle thoracic	10 (43.5)	31 (47.7)	
Lower thoracic	1 (4.3)	9 (13.8)	
Grading:			0.0356
G1/G2	22 (95.7)	49 (75.4)	
G3	1 (4.3)	16 (24.6)	
Clinical stage at diagnosis:			0.1190
I‐II	5 (21.7)	5 (7.7)	
III‐IV	18 (78.3)	60 (92.3)	
ASA:^b^			0.2140
1‐2	11 (47.8)	40 (64.5)	
3‐4	12 (52.2)	22 (35.5)	
Comorbidity:			
Liver disease	6 (26.1)	11 (16.9)	0.3656
Hypertension	9 (39.1)	17 (26.2)	0.2906
Diabetes	4 (17.4)	4 (6.2)	0.1982
Respiratory disease	7 (30.4)	13 (20.0)	0.3861
Cardiovascular disease	8 (34.8)	15 (23.1)	0.2825
Arteriosclerosis	2 (8.7)	7 (10.8)	0.9999
CT scheme:			0.4388
DDP±5FU	16 (69.6)	52 (80.0)	
Cetuximab‐based	1 (4.3)	4 (6.2)	
Platinum‐based	5 (21.8)	6 (9.2)	
Other scheme	1 (4.3)	3 (4.6)	

Note

Data are expressed as n(%) or

Abbreviations: yCR: clinical complete response. yPPD: clinical persistence or progressive disease. yPR: clinical partial response. yNC: stage disease did not change. yPD: progression of disease.

^a^ median(IQR).

^b^ Data not available in three patients.

### 3.3 Tumor immune infiltrate as predictor of ypCR after neoadjuvant therapy

**Figure 2:**
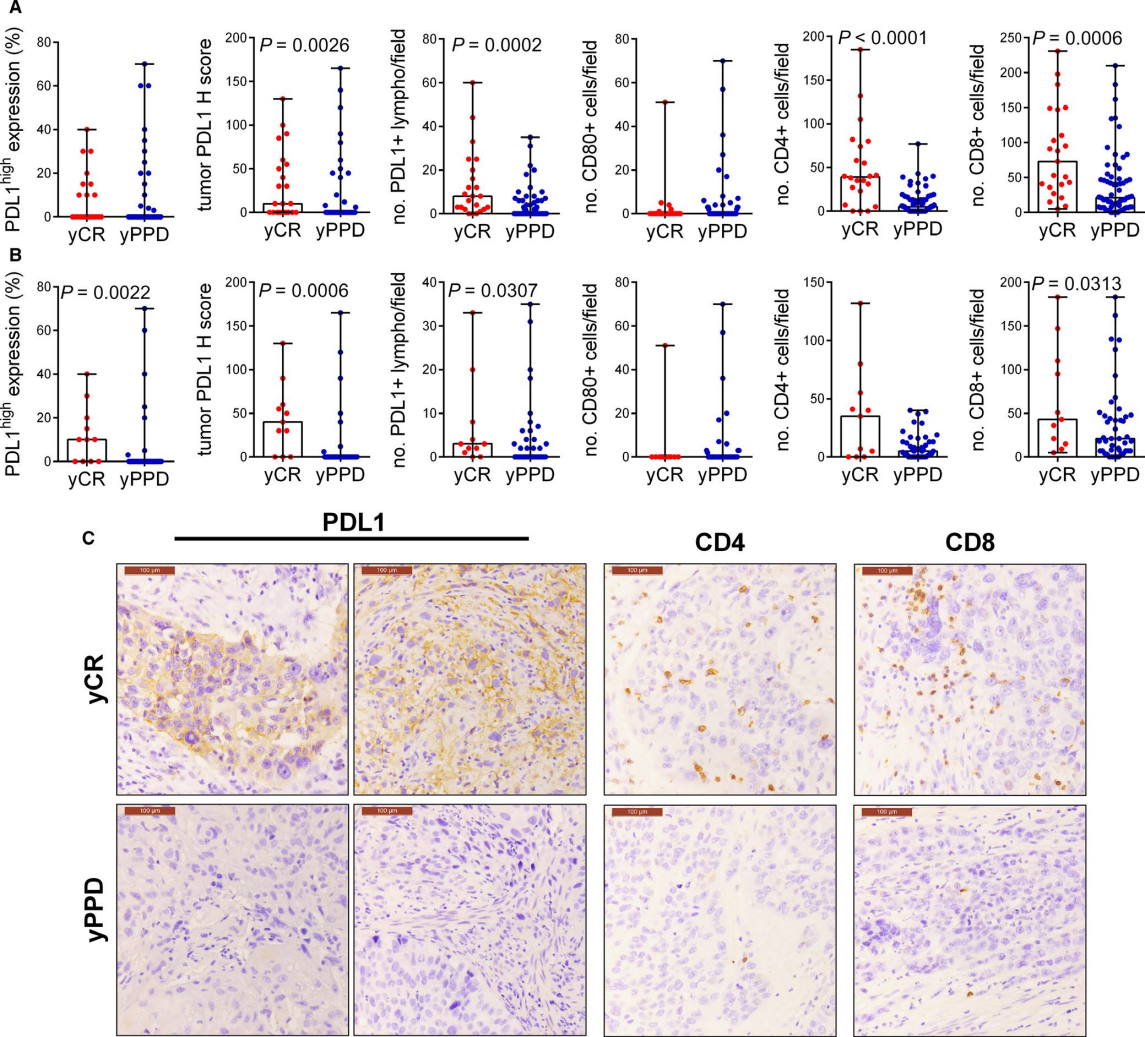
Immunological predictors of clinical outcome after neoadjuvant CT‐RT in esophageal squamous cell carcinoma. PD‐L1, CD80, CD4, CD8 expression in yCR and yPPD tumors according to response to neoadjuvant therapy in the whole cohort (A) and in clinical stage III‐IV tumors treated with DDP ± 5FU (B). Data are shown as. Min‐Max error bars and Mann‐Whitney test was used for the comparison. (C) Representative examples of immunohistochemical stainings of PD‐L1, CD4 and CD8 according to tumor responsiveness to neoadjuvant therapy (original magnifications 20×, scale bar = 100 µm).

Patients in ypCR group had higher levels of PD‐L1 expression either on tumor cells or in lymphocytes than patients in yPPD group (*P* = 0.004 and 0.0002, respectively). Moreover, in the ypCR group, CD4+ and CD8+ lymphocytes tumor infiltration was significantly higher than in yPPD group (*P* < 0.0001 and 0.0006, respectively). On the contrary, CD80 expression was not different in ypCR and yPPD groups (Figure 2A; Table S1A). The sensitivity sub‐analysis among patients who underwent DDP + 5FU CT scheme for clinical stage III‐IV SCC confirmed that higher levels of PD‐L1 expression either on tumor cells or in lymphocytes and CD8+ T cells infiltration were observed in ypCR group than in yPPD group. (Figure 2B; Table S1B).

**Figure 3:**
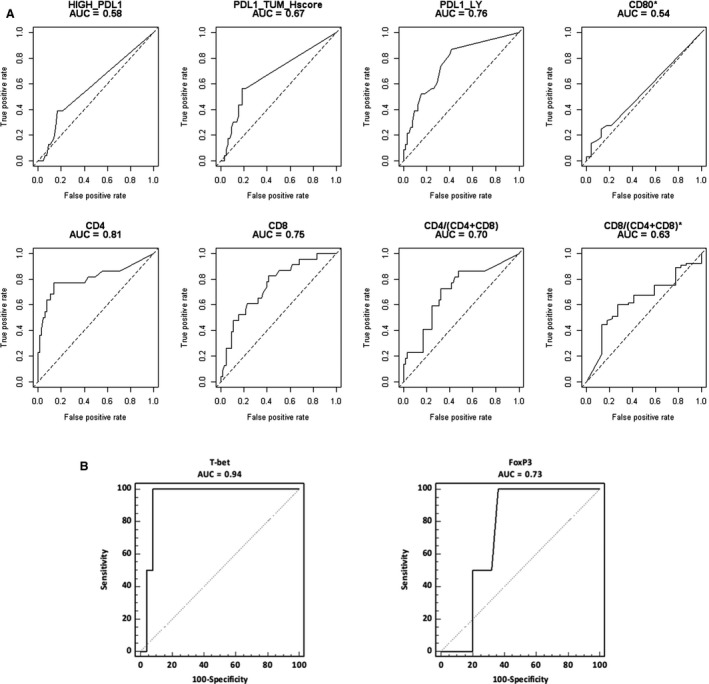
(A) ROC curves for yCR or *yPPD after neoadjuvant therapy; (B) ROC curves for FoxP3 and Tbet as predictor of yCR after neoadjuvant therapy. The accuracy of immunological markers as predictors of yCR was tested in the subgroup of patients with the ROC curve analysis.

ROC curve analysis revealed that PD‐L1 expression either on tumor cells or on lymphocytes had a moderate accuracy in predicting complete response (threshold value:>8; AUC = 0.67, *P* = 0.01 and threshold>0; AUC = 0.76, *P* = 0.001, respectively) (Figure 3A**)**. Similarly, CD4+ and CD8+ T cell infiltration showed an even better accuracy in predicting ypCR after neoadjuvant therapy (threshold>22; AUC = 0.81, *P* = 0.0001 and threshold>25; AUC = 0.75, *P* =0.0001, respectively) (Figure 3A). Finally, Tbet showed an optimal accuracy in predicting ypCR (threshold > 12; AUC = 0.94, *P* = 0.0002) (Figure 3B).

### 3.4 Tumor immune infiltrate as predictor of overall survival

**Figure 4:**
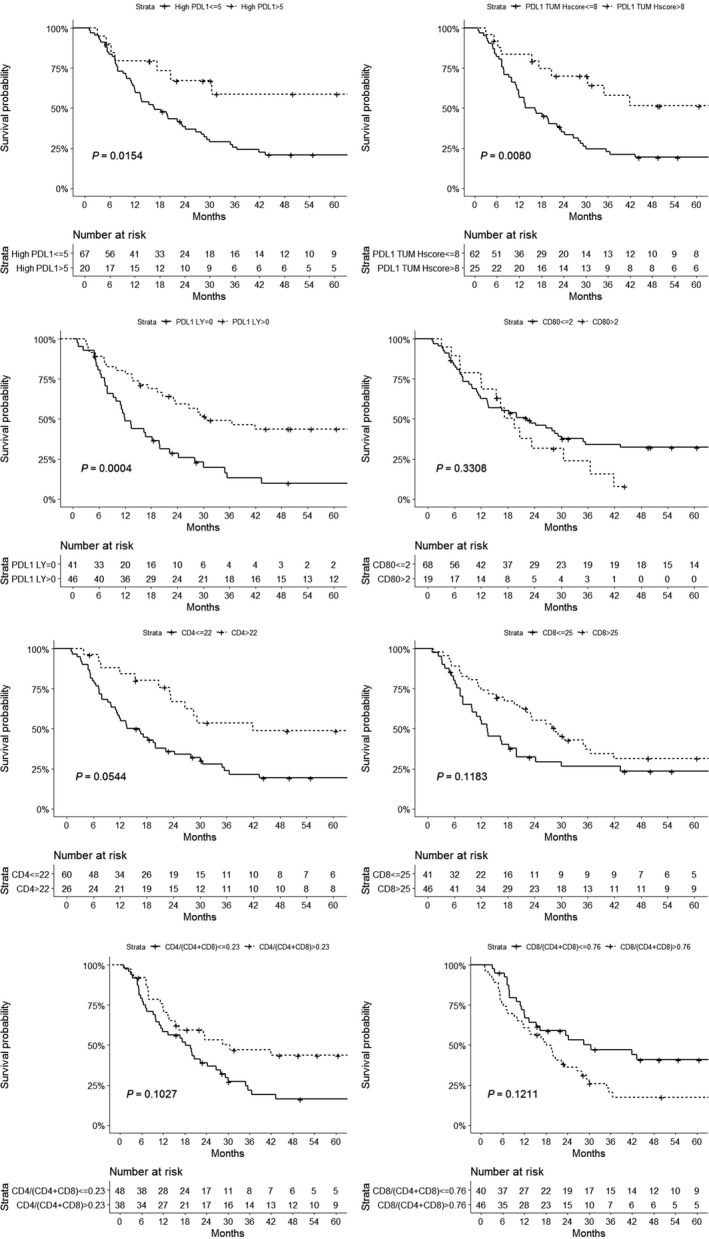
Tumor immune infiltrate as predictor of overall survival. The Kaplan‐Meier estimate was used to perform the survival analysis from the date of the initial diagnosis and the log‐rank test was used to compare the subgroup survival.

The association of tumor immune infiltrate markers and overall survival in SCC is shown in Figure 4 and Table S2. Patients with a low expression of PD‐L1 either on tumor cells or lymphocyte cells had a worse overall survival than those with high expression of PD‐L1 (*P* = 0.008 and 0.0004, respectively). Similarly, patients with a low infiltration of the tumor with CD4+ T cells had a significantly worse overall survival compared with those with high CD4+ infiltration (*P* =0.05). High infiltration of the tumor CD4+ T cells was associated with lower clinical stage at diagnosis (*P* = 0.006; Table S3).

### 3.5 Healthy mucosa immune infiltrate as predictor of persistence of complete response after neoadjuvant therapy

Healthy mucosa immune infiltrate did not show any significant association with recurrence after ypCR. Only high CD4+ infiltration might be associated with higher recurrence rate (*P* = 0.058) (Table S4). ROC curve analysis revealed poor discriminative performance of healthy mucosa immune infiltrate data regarding recurrence after ypCR. The association of immunological mucosal marker and recurrence after yCR in SCC are shown in Supplementary Figure 1.

## 4 DISCUSSION

In our esophageal SCC series, a higher CD4+ T cells tumor infiltration was significantly associated to a better overall survival. Similarly, in several studies, CD8+ and CD4+ T cell and CD57+ NK cells infiltration was correlated with better overall survival.^45^, ^46^ In addition, high CD8+/FOXP3+ and the CD8+/CD204+ ratios were significantly associated to a better prognosis after adjusting for clinicopathological factors.^47^ These data, taken together confirm the crucial role of T cells infiltration in the prognosis of SCC patients.

Since 2003, how the interplay between CD4+ and CD8+ T cells strongly correlates with esophageal SCC patients' prognosis has been shown,^48^ but the crucial question now is whether the immune infiltrate could predict the outcome after the neoadjuvant therapy. A recent article showed that the downregulation of CD4+CD25high+ Treg cells after chemotherapy might be a predictor for the outcome of chemotherapy in advanced esophageal carcinoma patients.^49^ Furthermore, in an interesting study that enrolled patients with esophageal SCC candidate to definitive CTRT, the clustering analysis on gene expression profiles clearly indicated that the increment of mRNA levels of cytotoxic T cells activation‐related genes is related to a better antitumor response in SCCs which show overexpression of these genes before CTRT.^50^ In the present study, CD8+ and CD4+ lymphocytes infiltrating the tumor before the neoadjuvant therapy were significantly higher in patients who achieved a yCR compared to those who had stable or progressed disease and CD4+ and CD8+ T cells infiltration showed a good accuracy in predicting yCR. Our data confirmed that sub population quantification of tumor infiltrating lymphocytes at IHC could be a clinically useful predictor for therapy response also in thoracic SCC.

Moreover, in our series, a sub‐analysis taking in account only patients who had stage III‐IV, SCC and who underwent DDP+5FU CT scheme confirmed a higher level of CD8+ T lymphocytes infiltrating the tumor in yCR group compared to yPPD group. Tsuchikawa et al. observed that neoadjuvant chemotherapy utilizing 5‐fluorouracil and cisplatin in SCC was useful to induce CD4 and CD8 T lymphocytes infiltration within the tumor microenvironment and to maintain HLA class I expression levels in combination with its direct cytotoxic effects.^51^ These data suggest that an elevated level of T cells infiltration within the tumor before the neoadjuvant therapy can be the substrate for a strong immune response induced by the liberation of cancer antigens due to tumor cells necrosis caused by the chemotherapy.

Nowadays the clinical significance of PD‐L1 expression within the tumor remains a debated topic. In colorectal cancer, PD‐L1 expression cancer defines three subsets of tumor immune microenvironments that can influence the response to therapy and the prognosis.^40^ Similarly, in advanced gastric cancer, PD‐L1 expression and CD8(+) T cell infiltration predict a favorable prognosis.^42^ Finally, in pulmonary adenocarcinoma, PD‐L1 expression appears to be a valid indicator of PD‐L1 status, showing significant correlation with low‐grade differentiation, lymphatic invasion and postoperative relapse‐free survival.^44^ On the other hand, in a recent study, PD‐L1 expression was associated with a significantly worse prognosis in patients with SCC undergoing esophagectomy or definitive radio‐chemotherapy.^52^, ^53^ In a different series, in patients with SCC who underwent surgery without preoperative therapy, high PD‐L1 expression was also associated with worse overall and relapse‐free survival.^51^, ^54^ On the contrary, in patients undergoing surgery alone, PD‐L1 expression was positively associated with a better prognosis.^55^ Similarly, PD‐L1 expression on immune cells was an independent prognostic factor for patients with esophageal SCC.^57^, ^58^ In our series, patients with a high expression of PD‐L1 either on tumor cells or on lymphocyte cells had a better overall survival than those with low expression of PD‐L1. In our opinion, high expression of PD‐L1 at baseline can be a signal of strong immune infiltration within the tumor. We hypothesize that this infiltrate is counterbalanced by a high PD‐L1 expression but the chemotherapy could unmask the cancer antigens, therefore the immune checkpoints will fall allowing a strong immune response.

In the last two years, PD‐L1 expression in esophageal SCC was largely explored but its role in neoadjuvant therapy response is largely unknown. PD‐L1 expression can be increased following treatment with 5‐FU in gastrointestinal cancer cell lines, suggesting alternative mechanisms to classic immune‐mediated upregulation.^59^ In our series, ypCR patients presented higher expression levels of PD‐L1 (both in tumor cells and in lymphocytes) than yPPD patients. The sensitivity analysis confirmed that among patients who underwent DDP+5FU CT scheme for clinical stage III‐IV SCC, higher levels of PD‐L1 expression (either on tumor cells or in lymphocytes) and CD8 T cells infiltration were observed in ypCR group than in yPPD group. The mechanism underlying this association remains unclear. However, in our series, PD‐L1 expression (either on tumor cells or on lymphocytes) had a good accuracy in predicting ypCR and it might be used as prognostic marker before initiating neoadjuvant therapy.

Moreover, the inhibition of the PD‐1/PD‐L1 axis in a neoadjuvant setting has recently shown good results in lung cancer. In fact, neoadjuvant nivolumab (antibody against PD‐1) was associated with few side effects, did not delay surgery, and induced a major pathological response in 45% of resected tumors.^60^, ^61^ Therefore, these studies and our results might be premises for a neoadjuvant therapy with check point inhibitor for locally advanced esophageal SCC.

Once the clinical CR is achieved, the main problem is how to predict the persistence of the response to CTRT. An accurate marker to persistence of yCR might be used to avoid unnecessary esophagectomy saving to patients' great risks, impaired quality of life and the costs of a major surgery procedure. In a recent multicenter series, among 593 patients who underwent neoadjuvant CTRT followed by esophagectomy, pCR was observed in 32% of patients but recurrence occurred in one third of these patients.^62^ In our series, recurrence after yCR occurred in 52.2% of patients and the most frequent site of recurrence was within the esophagus but the healthy mucosa immune infiltrate did not show any significant association with recurrence after yCR. Only high infiltration level of CD4+ T cell showed a trend to be associated to recurrence/relapse but the sub population analysis, to discriminate the role of Treg and Th1, was not possible due to the small sample size of the yCR group.

### 4.1 Limits of the study

In fact, the relatively small sample size and the consequent low power of the analysis are the main limitations of this exploratory study. Moreover, no public database on esophageal SCC which includes clinical/pathological response and molecular is currently available. Thus, larger, and hopefully multicenter, studies are needed to clearly identify predictors of recurrence after yCR at neoadjuvant therapy and to validate our results. On the other hand, the lack of previous study that tries to correlate the immune tumor microenvironment and response to neoadjuvant therapy make this study the necessary step to lay the foundation for these future multicenter large studies.

### 4.2 Conclusions

In conclusion, in our series, PD‐L1 expression and CD8+, CD4+ and Tbet+ lymphocyte rate were predictive of clinical CR after neoadjuvant CTRT for SCC of the thoracic esophagus with adequate accuracy. These preliminary observations might be used to plan further study aimed to identify reliable predictors of response to CTRT in esophageal SCC.

## CONFLICT OF INTEREST STATEMENT

None to declare.

## AUTHOR CONTRIBUTIONS

According to the CRediT taxonomy, the author contributions are as follows: Conceptualization: Matteo Fassan, Massimo Rugge, Francesco Cavallin, Carlo Castoro, Marco Scarpa, Ignazio Castagliuolo; Data curation: Vincenza Guzzardo, Andromachi Kotsafti, Melania Scarpa, Rita Alfieri, Matteo Cagol, Vanna Chiarion‐Sileni, Luca Maria Saadeh; Formal analysis: Matteo Fassan, Francesco Cavallin, Marco Scarpa; Funding acquisition: Carlo Castoro, Marco Scarpa; Investigation: Matteo Fassan, Massimo Rugge, Francesco Cavallin, Carlo Castoro, Marco Scarpa, Ignazio Castagliuolo; Methodology: Massimo Rugge, Ignazio Castagliuolo; Project administration: Marco Scarpa, Carlo Castoro; Resources: Marco Scarpa, Carlo Castoro; Software: not applicable. Supervision: Marco scarpa, Ignazio Castagliuolo, Massimo Rugge, Matteo Fassan; Validation: Vincenza Guzzardo, Andromachi Kotsafti, Melania Scarpa, Rita Alfieri, Matteo Cagol, Vanna Chiarion‐Sileni, Luca Maria Saadeh; Visualization: Melania Scarpa, Andromachi Kotsafti, Luca Maria Saadeh; Writing original draft: Matteo Fassan, Francesco Cavallin, Marco Scarpa; Writing review and editing:, Vincenza Guzzardo, Andromachi Kotsafti, Melania Scarpa, Matteo Cagol, Vanna Chiarion‐Sileni, Luca Maria Saadeh, Rita Alfieri, Ignazio Castagliuolo, Massimo Rugge, Carlo Castoro.
