# Supplementary material for: PD‐L1 expression, CD8+ and CD4+ lymphocyte rate are predictive of pathological complete response after neoadjuvant chemoradiotherapy for squamous cell cancer of the thoracic esophagus
Source: Cancer Med. 2019 Aug 20;8(13):6036–48. doi: 10.1002/cam4.2359 (PMC6792480; doi:10.1002/cam4.2359)
Supplement: Supplementary file 1 [file CAM4-8-6036-s001.docx]

**SUPPLEMENTARY MATERIAL**

**Supplementary results**

The expression of PDL-1 on tumor cells was not statistically different in patients with clinical stage I-II (median 0, IQR 0-48) and in those with clinical stage III-IV (median 0, IQR 0-10) (p=0.47). The expression of PDL-1 on lymphocyte cells was not statistically different in patients with clinical stage I-II (median 6, IQR 2-11) and in those with clinical stage III-IV (median 1, IQR 0-7) (p=0.07). The expression of PDL-1 on tumor cells was not statistically different in patients with G1/G2 tumor (median 0, IQR 0-11) and in those with G3 tumor (median 0, IQR 0-13) (p=0.49). The expression of PDL-1 on lymphocyte cells was not statistically different in patients with G1/G2 tumor (median 2, IQR 0-8) and in those with G3 tumor (median 0, IQR 0-2) (p=0.11). We think that the expression of PDL-1 on lymphocyte cells according to tumor stage or to degree of differentiation may be evaluated in larger samples.

Supplementary Table 1a. Immune infiltrate characterization according to response to neoadjuvant therapy

|  | yCR | yPPD | p-value |
| --- | --- | --- | --- |
| N pts | 23 | 65 | - |
| Percentage of high PD-L1 (2+; 3+) ^a^ | 0 (0-15) | 0 (0-0) | 0.1369 |
| PD-L1_ tumour cell_H score ^a^ | 10 (0-55) | 0 (0-0) | 0.0039 |
| PD-L1+ lymphocytes ^a^ | 8 (2-20) | 0 (0-5) | 0.0002 |
| CD80 ^a^ | 0 (0-0) | 0 (0-2) | 0.4874 |
| CD4 ^a^ | 39 (23-74) | 5 (0-13) | 0.0001 |
| CD8 ^a^ | 73 (36-147) | 21 (7-47) | 0.0006 |
| CD4/(CD4+CD8) ^a^ | 0.34 (0.20-0.46) | 0.16 (0.0-0.30) | 0.0055 |

Data are expressed as ^a^median(IQR) +ve cells number in 5 HPF (40x).

Supplementary Table 1b. Immune infiltrate according to the response to neoadjuvant therapy: sensitivity analysis on DDP±5FU (clinical stage III-IV)

|  | yCR | yPPD | p-value |
| --- | --- | --- | --- |
| N pts | 11 | 45 | - |
| Percentage of high PDL (2+; 3+) ^a^ | 10 (0-30) | 0 (0-0) | 0.0041 |
| PD-L1_ tumour cell_H score ^a^ | 40 (2-90) | 0 (0-0) | 0.0006 |
| PD-L1+ lymphocytes ^a^ | 3 (2-20) | 0 (0-4) | 0.0312 |
| CD80 ^a^ | 0 (0-0) | 0 (0-2) | 0.2790 |
| CD4 ^a^ | 35 (0-55) | 6 (0-16) | 0.1026 |
| CD8 ^a^ | 88 (21-110) | 21 (7-46) | 0.0330 |
| CD4/(CD4+CD8) ^a^ | 0.25 (0.0-0.46) | 0.16 (0.0-0.30) | 0.2878 |

Data are expressed as ^a^median(IQR) +ve cells number in 5 HPF (40x).

Supplementary Table 2. Overall survival according to clinical and immunological predictors

|  | 5-ys OS (%) | p-value |
| --- | --- | --- |
| All patients | 28% | - |
| yCR  yPR+yNC+yPD | 67%  13% | 0.0001 |
| Percentage of high PDL (2+; 3+)  ≤5  >5 | 21%  60% | 0.0154 |
| PD-L1_tumour cell_H score  ≤8  >8 | 16%  52% | 0.0080 |
| PD-L1+ lymphocytes  =0  >0 | 10%  43% | 0.0004 |
| CD80  ≤2  >2 | 26% at 3y)  34% (32 at 5y) | 0.3308 |
| CD4  ≤22  >22 | 19%  49% | 0.0544 |
| CD8  ≤25  >25 | 23%  32% | 0.1183 |
| CD4/(CD4+CD8)  ≤0.23  >0.23 | 16%  43% | 0.1027 |
| CD8/(CD4+CD8)  ≤0.76  >0.76 | 18%  40% | 0.1211 |

Supplementary table 3. Association between tumour stage and PDL-1 expression (both on tumor cells and lymphocyte cells), and infiltration of the tumor CD4+ T cells in 5 HPF (40x)

|  | Clinical stage at diagnosis | |  |
| --- | --- | --- | --- |
|  | I-II | III-IV | p-value |
| N of patients | 10 | 78 | - |
| Percentage of high PDL (2+; 3+)  ≤5  >5 | 7 (70.0)  3 (30.0) | 61 (78.2)  17 (12.8) | 0.6888 |
| PD-L1 tumour cell_H score  ≤8  >8 | 6 (60.0)  4 (40.0) | 57 (73.1)  21 (26.9) | 0.4607 |
| PD-L1+ lymphocytes  =0  >0 | 2 (20.0)  8 (80.0) | 39 (50.0)  39 (50.0) | 0.0974 |
| CD4  ≤22  >22 | 3 (30.0)  7 (70.0) | 69 (75.6)  19 (24.4) | 0.0066 |

Data are expressed as n(%) of patients with a determinate number of positive cells in 5 HPF (40x) and comparison were performed with Fisher exact test. .

Supplementary table 4. Recurrence/relapse after yCR and immunosurveillance data (groups according to ROC threshold values). Non-parametric combination test was used for comparisons.

| Immunological markers | N | Recurrence/relapse | p-value ^a^ |
| --- | --- | --- | --- |
| *PD1*  *<=39*  *>39* | *16*  *7* | *10 (62.5)*  *2 (28.6)* | *0.1930* |
| *Percentage of high PDL (2+; 3+)*  *=0*  *>0* | *14*  *9* | *9 (64.3)*  *3 (33.3)* | *0.2138* |
| *PD-L1_ tumour cell_H score*  *<=40*  *>40* | *16*  *7* | *10 (62.5)*  *2 (28.6)* | *0.1930* |
| *PD-L1+ lymphocytes*  *>6*  *<=6* | *12*  *11* | *7 (58.3)*  *5 (45.5)* | *0.6843* |
| *CD80*  *=0*  *>0* | *18*  *5* | *10 (55.6)*  *2 (40.0)* | *0.6403* |
| *CD4 ^c^*  *>80*  *<=80* | *4*  *18* | *4 (100.0)*  *7 (38.9)* | *0.0580* |
| *CD8*  *<=15*  *>15* | *3*  *20* | *3 (100.0)*  *9 (45.0)* | *0.2174* |
| *CD4/(CD4+CD8) ^c^*  *>0.25*  *<=0.25* | *14*  *8* | *8 (57.1)*  *3 (37.5)* | *0.5294* |
| *CD8/(CD4+CD8) ^c^*  *<=0.69*  *>0.69* | *13*  *9* | *8 (61.5)*  *3 (33.3)* | *0.3870* |

*Data are expressed as n (%) of patients with a determinate number of positive cells in 5 HPF (40x). ^a^NPC test. ^c^Data not available in one patient.*
